# Expression of DNMTs and H3K9ac in Ameloblastoma and Ameloblastic Carcinoma

**DOI:** 10.3389/froh.2021.751162

**Published:** 2021-10-26

**Authors:** Gleyson Kleber do Amaral-Silva, Thayná Melo de Lima Morais, Vivian Petersen Wagner, Manoela Domingues Martins, Eduardo Rodrigues Fregnani, Fernando Augusto Soares, André Caroli Rocha, Helder Rabelo Pontes, Alan Roger Santos-Silva, Pablo Agustin Vargas

**Affiliations:** ^1^Department of Oral Diagnosis, Piracicaba Dental School, University of Campinas, Piracicaba, Brazil; ^2^Department of Pathology, School of Dentistry, Federal University of Rio Grande do Sul, Porto Alegre, Brazil; ^3^Department of Oral Medicine, Sírio-Libanês Hospital, São Paulo, Brazil; ^4^Medical School, Clinics Hospital, University of São Paulo, São Paulo, Brazil; ^5^Service of Buccal Pathology, João de Barros Barreto University Hospital, Federal University of Pará, Belém, Brazil

**Keywords:** ameloblastoma, DNA methylation, epigenetic, methyltransferases, histone modifications, recurrences

## Abstract

**Objectives:** DNA methyltransferases (DNMTs) and the histone modification H3K9ac are epigenetic markers. This study aimed to describe the immunohistochemical expression of DNMT1, DNMT3A, DNMT3B, and H3K9ac in the dental follicle (DF), ameloblastoma (AME), and ameloblastic carcinoma (AC), correlating these expressions with the recurrence and aggressive behavior in ameloblastoma.

**Study Design:** Immunohistochemical reactions were performed in 10 human DFs, 38 ameloblastomas, and 6 AC samples. Another 59 ameloblastomas assembled in a tissue microarray were used to compare the immunoexpression with the clinical, radiographic, and histopathological characteristics and the presence of BRAFv600e mutation. Each slide was digitized as a high-resolution image and quantified by Aperio ScanScope Nuclear V9 software. All statistical analyzes were performed using GraphPad Prism statistical software.

**Results:** DNMT3B expression was higher in ameloblastomas than in the DFs, while the AC overexpressed all proteins. The ameloblastomas with BRAFv600e mutation, vestibular/lingual, or vestibular/palatine bone cortical disruption and maxilla involvement showed DNMT1 overexpression, while recurrent cases had high DNMT3B levels.

**Conclusions:** DNA methylation and histone modification might play a role in the development, clinical aggressiveness, and recurrence rates of ameloblastoma, such as the progression to AC. Further investigation about gene methylations in ameloblastomas is needed to better understand its relationship with aggressiveness and recurrence.

## Introduction

Epigenetic events are mechanisms responsible for controlling gene expression, playing a role in several physiological and pathological functions without altering the genome sequence [1]. DNA methylation is one of these mechanisms, characterized by methyl group addition in the cytosine-phosphate-guanine (CpG) dinucleotide—mostly found in the gene's promoter region. Once present, the methyl group transforms the cytosine in 5-methylcytosine (5mC), which ends up blocking the transcriptional machinery to engage in the gene's promoter area, causing gene silencing. This reaction is possible due to the DNA methyltransferase's (DNMTs) catalytic activity, developed by the DNMT1, DNMT3A, and DNMT3B proteins [2–4]. DNA methyltransferase 1 is responsible for conserving the DNA methylation pattern, copying the CpG methylation status in the parental DNA strand to the daughter DNA strand during replication. On the other hand, DNMT3A and DNMT3B are responsible for creating the *de novo* methylation pattern and can inactivate new gene expressions [3, 5–7]. The dynamism in this epigenetic control is possible due to the demethylation antagonist action, catalyzed by the ten-eleven translocation (TET) proteins, which removes the methyl group from the 5mC [8].

Histone modifications are another epigenetic mechanism intensely studied, especially the acetylation on histone H3 at lysine 9 (H3K9ac) [9, 10]. The histone acetyltransferase (HAT) enzymes attach the acetyl group to the histones, reducing the differential charges between the histones (positive) and the DNA (negative). This event causes chromatin decompression, allowing previously inaccessible genes to be exposed to transcriptional factors, thus triggering their expression [11–13]. Among different kinds of histone modifications, H3K9ac is recognized as essential to gene transcription activation [14].

Epigenetic mechanisms are crucial in physiologic functions but are also key regulators in pathologic conditions. Histone acetylation, for example, has been previously associated with head and neck squamous cell carcinoma aggressiveness [15]. Moreover, epigenetic events are currently considered a potential therapeutic target through the use of epi-drugs [16, 17], with promising results achieved in different types of head and neck tumors such as squamous cell carcinoma [18] and salivary gland tumors [19]. In odontogenic lesions, the clinical relevance of epigenetic mechanisms remains uncertain. Therefore, we aimed to investigate DNMT1, DNMT3A, DNMT3B, and H3K9ac immunohistochemical expression in the most prevalent and locally aggressive benign odontogenic tumor, ameloblastoma, and compare this with its malignant counterpart, ameloblastic carcinoma (AC). Additionally, we investigated the relation of these DNMTs and H3K9ac with the prognosis and recurrence of conventional ameloblastoma.

## Materials and Methods

### Samples

The Ethical Committee of the research institution approved this study (79140917.9.0000.5418). The informed consent was obtained for all patients and the study was performed in accordance with the Declaration of Helsinki. Firstly, formalin-fixed paraffin-embedded specimens of 10 dental follicles (DF), 38 conventional ameloblastomas (AME), and six AC were used to compare the quantitative differences among DNMT1, DNMT3A, DNMT3B, and H3K9ac positive cells. Secondly, 59 ameloblastomas assembled in a TMA block (AMEtma group) with duplicated 1.0-mm-diameter cases—previously used by Fregnani et al. [20]—were used to show the positive cells quantified in each antibody with categorical variables (BRAFv600e, recurrence, vestibular/lingual, or vestibular/palatine and basal bone cortical integrity, radiographic features, gender, and tumor location). Table 1 shows a detailed description of the samples. All paraffin blocks underwent 3-μm sections in silanized glass slides for the immunohistochemistry reactions. This study used the DF as control tissue due to the gene expression similarity between the dental epithelium and ameloblastoma [21].

**Table 1:** Multiple comparisons among groups for each protein (Kruskal–Wallis followed by Benjamini, Krieger and Yekutieli *post-hoc* tests).

	**Kruskal–Wallis**	**Follicle × AME**	**Follicle × AC**	**AME × AC**
	***P*** **-value**	***P*** **-value (diff.)**	***P*** **-value (diff.)**	***P*** **-value (diff.)**
DNMT1	0.0448^*^	0.7418	0.0243^*^(−17.97)	0.0175^*^(−16.15)
DNMT3A	0.0245^*^	0.5676	0.0118^*^(−20.11)	0.0113^*^(−16.89)
DNMT3B	0.0001^*^	0.0001^*^(−21.72)	0.0003^*^(−28.07)	0.1253
H3K9ac	0.0018^*^	0.4258	0.0134^*^(−20.10)	0.0004^*^(−24.55)

* *Statistically significant*.

### Immunohistochemistry and Digital Analysis

After deparaffinization and hydration steps, the antigen retrieval was performed in an electric pressure cooker for 15 min with Tris-EDTA buffer (pH 9.0). Endogenous peroxidase inhibition was achieved with 3% aqueous hydrogen peroxidase for 20 min at room temperature, followed by washes with 10 mM phosphate-buffered saline (pH 7.4) for 5 min. The primary antibodies incubated for 2 h were DNMT1 (ab134148, dilution 1:200, Abcam, Cambridge, UK), DNMT3A (NBP1-85961, dilution 1:100, Novus Biologicals, Newcastle, UK), DNMT3B (ab227883, dilution 1:200, Abcam) and H3K9ac (C5B11, dilution 1:500, CellSignaling, Danvers, MA, USA). The reactions were visualized using ADVANCE™/HRP (Dako, Carpinteria, CA, USA), exposure to diaminobenzidine tetrahydrochloride (DAB, Sigma, St. Louis, MO, USA), and Carazzi haematoxylin. The positive control used for DNMT1, DNMT3A, and H3K9ac was oral squamous cell carcinoma, while DNMT3B was normal thyroid tissue. The negative controls were obtained by omitting the primary antibodies.

Each immunohistochemical slide was scanned with an Aperio ScanScope CS Slide Scanner (Leica Microsystems, Wetzlar, Germany), creating high-resolution images in.svs format. The positive cell quantification for each case used the Nuclear V9 algorithm (Leica): in the DF, AME, and AC samples, 10 standardized rectangles—each with a 7.5-μm^2^ area—were allocated in the odontogenic epithelium. In AMEtma samples, the odontogenic epithelium was selected using the “pen” and “negative pen” software tools in each core/case. The software input parameters were: averaging radius: 1.5; curvature threshold: 1; minimum nuclear size: 1; maximum nuclear size: 200; minimum roundness: 0.1; minimum compactness: 0.1; minimum elongation: 0.2; clear area intensity: 240; lower intensity threshold: 0; upper intensity threshold: 255; and a positive intensity threshold ranging from 0 (strong) to 210 (weak and moderate).

### Statistical Analysis

The data analyzes was performed using the statistical software GraphPad Prism (version 8.0.0, GraphPad Software, San Diego, California, USA). All tests used a 95% confidence interval, two-tailed and α = 0.05 parameters. The Shapiro–Wilk and D'Agostino–Pearson tests were used when the groups presented *n* < 20 and *n* ≥ 20 samples, respectively. The Kruskal–Wallis test and Benjamini, Krieger and Yekutieli *post-hoc* tests were performed for the DF, AME, and AC groups. The unpaired *t*-test, *t*-test with Welch's correction (when Levene's test showed variance heterogeneity), and Mann–Whitney test were used for the AMEtma samples to compare the positive cell rates with different categorical variables; when statistically significant, the ROC curve was used to identify the cut-off point. Finally, the Fisher exact test and Kaplan–Meier curve for recurrence-free survival (RFS) were performed.

## Results

**Figure 1:**
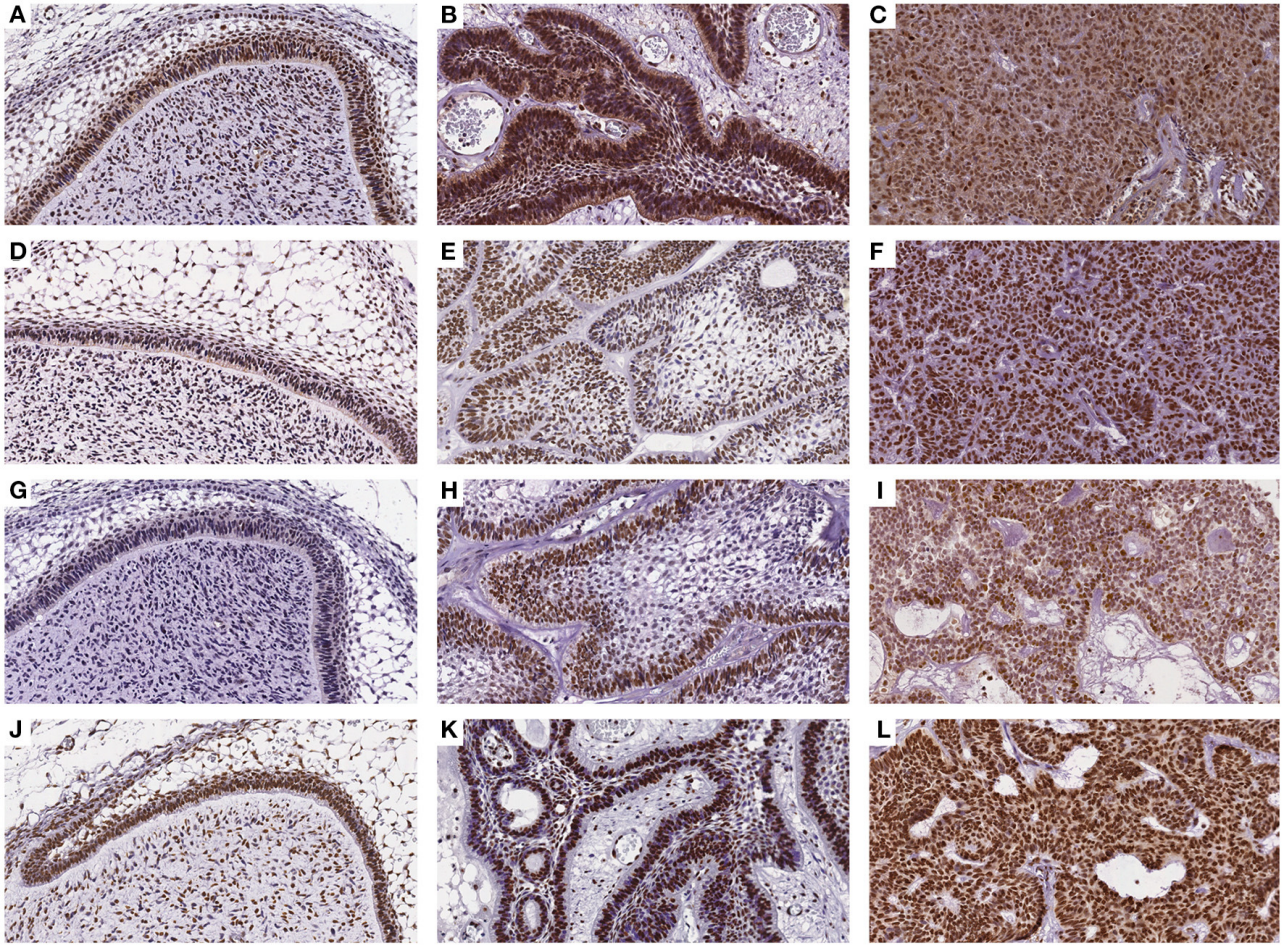
Immunohistochemical reaction for DNMT1, DNMT3A, DNMT3B, and H3K9ac antibodies in DF **(A,D,G,J)**, AME **(B,E,H,K)**, and AC samples **(C,F,I,L)**, respectively. (DAB, 200×).

**Figure 2:**
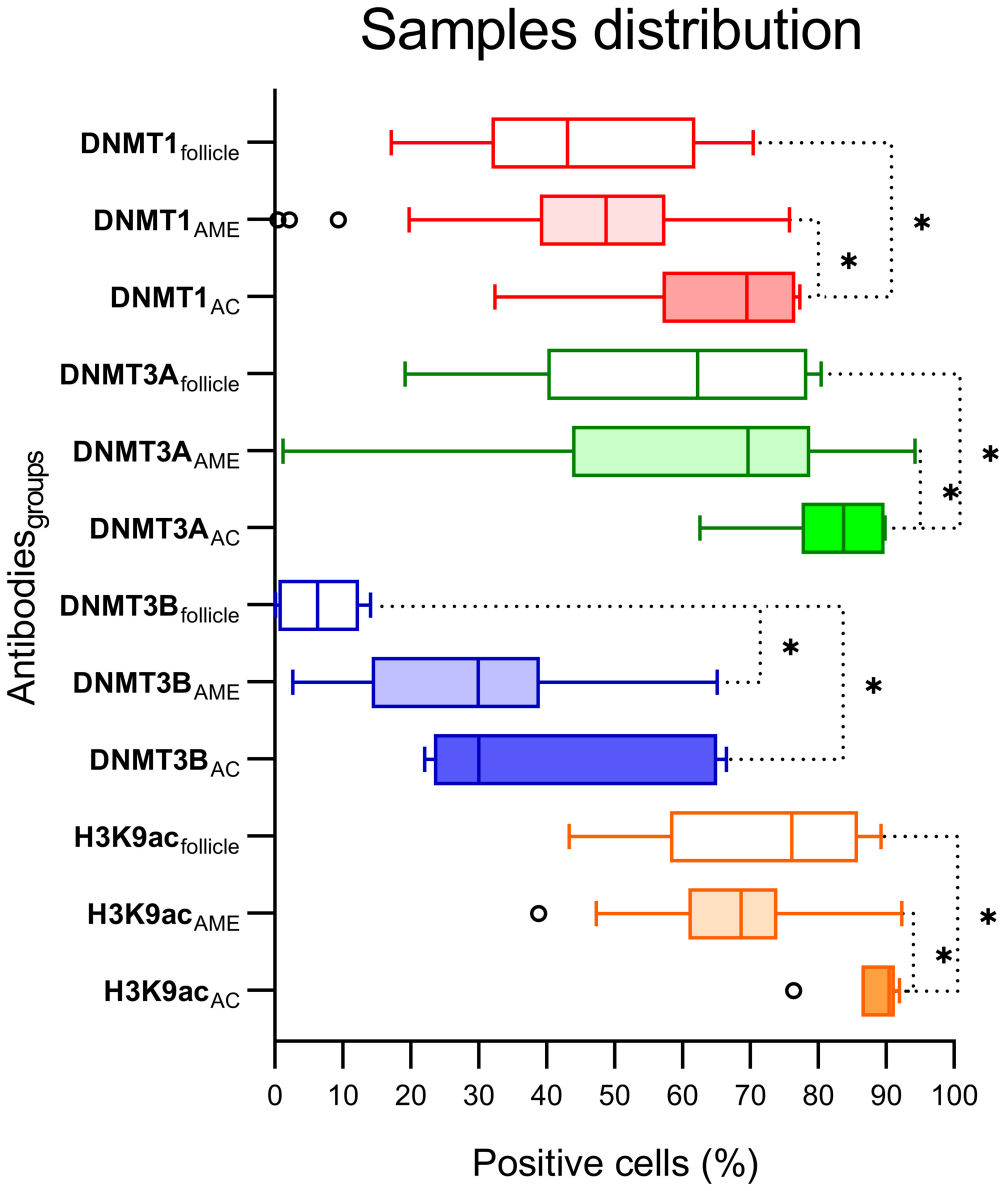
Graphic distribution of positive cells (%) for each antibody and DF, AME, and AC groups. ^*^Statistical difference between groups comparison.

The immunohistochemical stains for each antibody in the DF, AME, and AC samples are shown in Figure 1. In the DF group, high DNMT1 and DNMT3A expression were scattered in the ameloblastic epithelium, stellate reticulum, dental papilla, and connective tissue. On the other hand, the DNMT3B nuclear stain was predominantly negative, except in some cells located in the dental lamina of bud and cap phases and endothelial and osteoblast-like cells. This relative absence of DNMT3B is expected in normal tissue, except in the thyroid, testes and bone marrow6. In the AME and AC samples, DNMT1 and DNMT3A were strongly and diffusely expressed in all cell layers, regardless of the predominant histological pattern. Surprisingly, DNMT3B expression was increased in ameloblastic cells of the AME and AC group, mainly in the plexiform histological pattern. In contrast, expression in the follicular pattern was variable, showing nuclear positivity in the peripheral layer and a cytoplasmic stain in some acanthomatous areas. The DNMT3B stain in AME and AC connective tissue was similar to the DF group. The nuclear H3K9ac expression was present in epithelial and mesenchymal cells of all groups. The number of positive cells in each sample category and the results of the comparative tests are illustrated in Figure 2 and Table 1, respectively. The AME showed more DNMT3B positive cells than did DF (*p* = 0.0001), but similar to the AC group (*p* = 0.3581). On the other hand, AC showed higher immunoreactivity for all the other proteins compared to DF and AME.

The cell positivity rates in the AMEtma samples were compared among the different categorical variables, aiming to evaluate the association of the epigenetic mechanisms investigated with tumor recurrence and aggressive behavior. Supplementary Figure 1 shows the data distribution, and the comparative tests are summarized in Table 2. There were more DNMT1 positive cells in cases with BRAFv600e mutation than wild-type BRAF (wdBRAF; *p* = 0.0114), located in the maxilla than the mandible (*p* = 0.0072), and with vestibular/palatine or vestibular/lingual bone cortical ruptured than intact (*p* = 0.0066). The recurrent cases showed more DNMT3B positive cells than did non-recurrent cases (*p* = 0.0477). Others variables such as gender, radiographic features, and basal cortical integrity showed no statistical significance.

**Table 2:** Comparison among the presence of BRAFv600e mutation and clinical and radiographic features in the AMEtma group.

	**DNMT1**	**DNMT3A**	**DNMT3B**	**H3K9ac**
	***P*** **value (diff.)**	***P*** **value**	***P*** **value (diff.)**	***P*** **value**
**BRAF**				
BRAF × BRAFv600e	0.0114^*^(−12.14 ± 4.617)^a^	0.5038^a^	0.1553^a^	0.1464^b^
**Recurrence**				
Non-recurrence × Recurrence	0.0709^a^	0.2205^b^	0.0477^*^(−9.831 ± 4.841)^a^	0.1213^a^
**Gender**				
Female × Male	0.7864^a^	0.798^a^	0.8476^a^	0.2754^a^
**Radiographic feature**				
Multilocular × Unilocular	0.5669^a^	0.5941^a^	0.7882^a^	0.0689^a^
**Tumor site**				
Maxilla × Mandible	0.0072^*^(14.82)^b^	0.0878^a^	0.807^b^	0.2672^a^
**Cortical integrity (VLVP)**				
Intact × Disrupted	0.0066^*^(−11.28 ± 3.934)^c^	0.3218^a^	0.6877^a^	0.2912^a^
**Cortical integrity (B)**				
Intact × Disrupted	0.179^a^	0.9519^a^	0.8061^b^	0.3807^a^

* *Statistically significant; VLVP, vestibular/lingual or vestibular/palatine; B, basal*;

a *unpaired t-test*;

b *Mann–Whitney test*;

c *Welch's t-test; diff: difference*.

**Figure 3:**
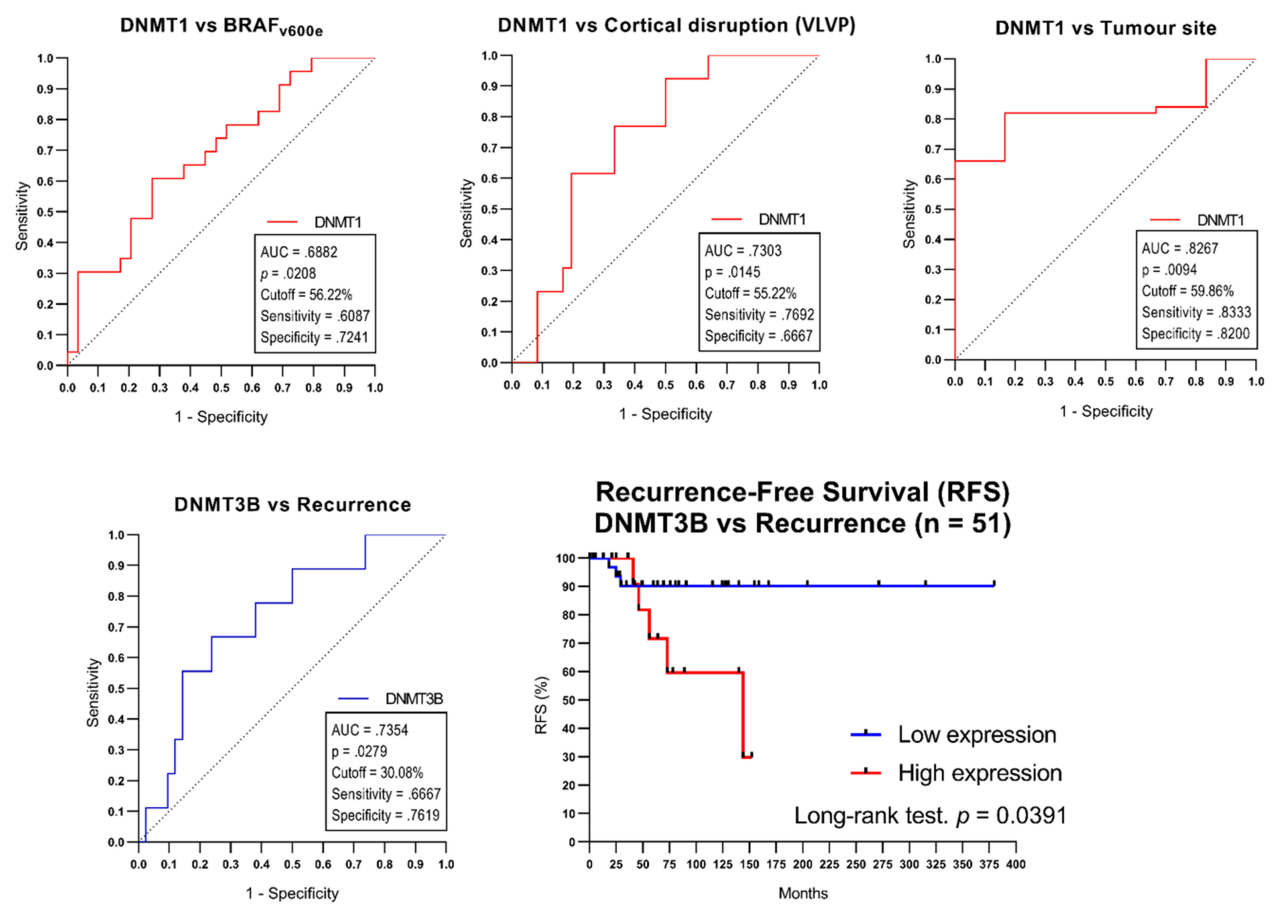
The cut-off points determined by ROC curve tests only in categorical variables comparison with a significate difference in the AMEtma group. The Kaplan-Mayer curve for Recurrence-Free Survival (RFS) for different DNMT3B expressions in the AMEtma group. VLVP, vestibular/lingual or vestibular/palatine bone cortical.

The DNMT1 cut-off points for the “wdBRAF-vs-BRAFv600e,” “maxilla-vs.-mandible,” and “intact-vs.-ruptured vestibular/palatine or vestibular/lingual bone cortical” variables were 56.22, 59.86, and 55.22%, respectively. The DNMT3B cut-off point was 30.08% for the “non-recurrence-vs.-recurrence” variable. The sensitivity, specificity, *p*-value, and area under the curve (AUC) are presented in Figure 3. The Fisher exact and odds ratio tests (Table 3) revealed that patients with cell positivity above the cut-off points had an increased risk of presenting BRAFv600e mutation, recurrence, vestibular/palatine, or vestibular/lingual bone cortical rupture, and maxilla involvement (4.1, 6.2, 6.7 and 18.3 more times, respectively). The RFS curve (Figure 3) showed a significant difference between patients above (high expression) and below (low expression) the DNMT3B cut-off point (*p* = 0.0391; log-rank test), corroborating that high DNMT3B expression is associated with poor RFS in AME.

**Table 3:** Contingency analyses (Fisher exact test) in significant variables using the cut-off point.

**Antibody (Cut-off%)**	**Low expression**	**High expression**	**Fisher exact test** ***P*****-value**	**Odds ratio (95% CI)**
**DNMT1 (56.22%)**				
BRAF	21	8	0.024^*^	4.083 (1.273–13.02)
BRAFv600e	9	14		
**DNMT3B (30.06%)**				
Non-recurrence	31	10	0.0219^*^	6.2 (1.434–24.81)
Recurrence	3	6		
**DNMT1 (59.86%)**				
Mandible	33	9	0.0058^*^	18.33 (2.303–220.4)
Maxilla	1	5		
**DNMT1 (55.22%)**				
(VLVP) intact	24	12	0.0097^*^	6.667 (1.621–24.85)
(VLVP) disrupted	3	10		

* *Statistically significant; VLVP, vestibular/lingual or vestibular/palatine bone cortical*.

## Discussion

The study of epigenetic events in odontogenic benign and malignant neoplasms can contribute to deepening our understanding of the dynamic and complex mechanisms involved with tumor development and progression. It also has the potential to reveal aggressive signatures and ultimately indicate promising therapeutic targets. To the best of our knowledge, this is the first study to correlate the expression of DNMTs and H3K9ac with clinical and radiographic features, recurrence, and BRAFv600e mutation in AME. Interestingly, our results suggest that among the epigenetic mechanisms evaluated, changes in DNA methylation, through DNMT activation, have a more significant role in AME growth and aggressiveness compared to histone acetylation. Moreover, DNA hypermethylation triggered by DNMT overexpression in AME could induce the silencing of important regulatory and tumor suppressor genes, such as cell cycle genes [22–24], apoptosis [25], matrix metalloproteinases [26], LINE-1 [27], and the mismatch repair genes MSH2 and MSH6 [28]. In AC samples, only the p16 gene was investigated and shown hipermethylation [22, 29]. The epigenome is still a poorly researched field in AME and AC. The few studies of hypermethylated genes in these tumors, together with the results in the present study, aim to stimulate further research in this area.

Because few studies have investigated these specific epigenetic mechanisms in odontogenic tumors, we thought it was important to correlate our finding with other relevant oral neoplasms. For example, DNMT3A and DNMT3B are increased in lip and oral carcinogenesis [30–32], and high levels of DNMT1 can also be seen in oral lichen planus [33]. These results corroborate with our findings, suggesting a hypermethylated profile in different oral pathologies. On the other hand, some studies have found that DNMT1 expression was similar among normal salivary gland tissue and benign and malignant salivary gland tumors [34, 35]. However, Shieh et al. found that mucoepidermoid carcinoma (MEC) without DNMT1 expression had longer survival rates than MEC patients with DNMT1 immunoreactivity (mean 71.9 and 31.9 months, respectively) [35]. Our results were somewhat similar in demonstrating the biomarker potential of DNMT proteins in oral neoplasms because AME patients had a lower risk of recurrence in the absence of DNMT3B. Interestingly, previous studies have identified significant differences in the percentage of cells expressing H3K9ac among benign and malignant salivary gland tumors, with malignancy correlated to a hypoacetylated profile [36], and have also determined that in oral squamous cell carcinoma, hypoacetylation is associated with chemoresistance, cancer stem-cell accumulation, and poor prognosis [15, 37, 38]. In the present study, no such significant results were encountered, which suggests that histone acetylation might not play such an important role in odontogenic tumors.

Concerning odontogenic cysts and tumors, only two previous studies have investigated DNMT expression (only in reactive or benign entities), and as far as we know, no previous reports exist on H3K9ac expression in this group of lesions. Gomes et al. were the first to describe DNMT1 and DNMT3A immunohistochemical expression in AME samples. Considering nuclear-cytoplasmic reactions, the authors found that all cases showed more than 25% DNMT1 positive cells, while 13 out of 16 cases showed a DNMT3A cytoplasmic reaction [39]. Guimarães et al. also found nuclear-cytoplasmic positivity for DNMT1 (higher than 50% of cells), DNMT3A (lower than 20%), and DNMT3B (higher than 50%) [40]. Although we also observed cytoplasmic stains in our samples, we believe these are background than a genuine reaction. In this present study, our AME samples showed 46.05, 60.83, 28.97, and 67.84% of mean nuclear positivity for DNMT1, DNMT3A, DNMT3B, and H3K9ac, respectively. Due to its rarity, we collected only six AC samples. However, the mean nuclear positivity for DNMT1, DNMT3A, DNMT3B, and H3K9ac, were, respectively, 65.07, 82.03, 39.56, and 88.37%, representing the first report of these proteins in a malignant odontogenic tumor. Our results are in agreement with previous studies [39, 40], which suggests that epigenetic dysfunctions could play a role in odontogenic lesion development. The increase of H3K9ac in AC suggests that previously suppressed oncogenes could now be transcribed due to DNA access by histone hyperacetylation. In addition, tumor suppressor genes could have their expression reduced by the hypermethylation caused by DNMT overexpression. Combined, these epigenetic events could favor the malignization of odontogenic cells.

Our study was novel in investigating the correlation between DNMT and H3K9ac expressions with clinical and radiographic features, recurrence, and BRAFv600e mutation in AME. Our results suggest that tumors with DNMT1 and DNMT3B overexpression can be more locally aggressive and have an increased risk of recurrence, respectively. Furthermore, the ameloblastoma aggressiveness is also associated with the BRAFv600e mutation [20]. Therefore, the relationship BRAFv600e-DNMT1 found in our study sharing this common tumor feature. Abnormal DNA methylation patterns can cause tumor suppressor gene silencing [41], which could then trigger the aggressive behavior observed herein. This finding is in accordance with what has been previously observed in other human neoplasms, such as oral cancer [42], esophageal squamous cell carcinoma [43], melanoma [44], diffuse large B-cell lymphomas [45], hepatocellular cancer [46], and gastric cancer [47], showing DNMT1 and DNMT3B overexpression associated with poor prognosis. We identified no association between immunohistochemical H3K9ac expression and tumor features. However, additional studies are needed to investigate the impact of histone modifications in odontogenic tumors.

The management of AME is currently surgical-based, which can result in important morbidity, especially for relatively large lesions. Moreover, surgical treatment of tumor relapse can be extremely challenging. Due to the high prevalence of MAPK-pathway alterations in AME, most efforts of target therapy for this neoplasm are associated with mechanisms involved with this pathway. Many reports exist of AME cases successfully treated with BRAF kinase or inhibitors, yet this treatment option still faces may challenges mostly associated with toxicity and acquired resistance [48]. Moreover, AC is a malignant tumor that lacks a specific systemic therapy and is mostly managed by surgical resection with adjuvant radiotherapy or conventional chemotherapy if needed [49]. Recently, the use of a new group of drugs, recognized as epi-drugs, has emerged as a promising therapeutic option in target therapy aiming to restore deregulated epigenetic mechanisms. Inhibitors of histone deacetylases (HDACi) or DNA methyltransferases (DNMTi) are among this new class of drugs, and some have already been approved by the Food and Drug Administration for some specific type of cancers [50]. Based on our results, we believe it would be more valuable to investigate the effects of DNMTi such as azacytidine and decitabine in AME and AC cells. The overexpression of all DNMTs in AC suggests an important deregulation in odontogenic malignant cells' methylation status. However, some limitations of epi-drugs treatment are notable, such as poor bioavailability and toxic adverse effects. The use of lower doses to minimize side-effects can be then compensated by associating with other agents, usually an immunotherapy drug [50]. Moreover, it is important to highlight our AC sample size as a limitation of the study, inherent to the rarity of this tumor. Our results suggested a possible relevance of DNMTs and H3K9ac in AC development, but further studies with a more representative sample of malignant odontogenic tumors are necessary to confirm this hypothesis.

In conclusion, AME growth and biological features could be related to an abnormal DNA methylation process. DNMT1 and DNMT3B may be useful epigenetic markers in this tumor, indicating their aggressiveness, BRAFv600e mutation, and recurrence chances. This field deserves to be expanded by evaluating tumor suppressor gene methylation status in AME. H3K9ac immunohistochemical expression had no involvement in AME features or development. However, we recognize that further research is needed to confirm or adjust our cut-off points. Moreover, different molecular and cellular methods are needed to assess histone modification profiles in odontogenic tumors.

## Data Availability Statement

The raw data supporting the conclusions of this article will be made available by the authors, without undue reservation.

## Ethics Statement

The studies involving human participants were reviewed and approved by Ethics Committee of Piracicaba Dental School—UNICAMP (protocol number/approval: 79140917.9.0000.5418). Written informed consent to participate in this study was provided by the participants' legal guardian/next of kin.

## Author Contributions

GA-S, TM, and VW performed the experiments and the data analyses. GA-S drafted the manuscript and generated the text and figures. MM, EF, FS, AR, HP, and AS-S critically revised the manuscript. PV provided leadership for the project. All authors contributed to the final manuscript.

## Funding

We are grateful to the São Paulo State Research Foundation (FAPESP) for a grant support (process number 2017/08995-2).

## Conflict of Interest

The authors declare that the research was conducted in the absence of any commercial or financial relationships that could be construed as a potential conflict of interest.

## Publisher's Note

All claims expressed in this article are solely those of the authors and do not necessarily represent those of their affiliated organizations, or those of the publisher, the editors and the reviewers. Any product that may be evaluated in this article, or claim that may be made by its manufacturer, is not guaranteed or endorsed by the publisher.

## Supplementary Material

Supplementary Figure 1 A AMEtma core sample before **(A)** and after application of Nuclear V9 software **(B)**, with DNMT3A reaction (DAB, ScanScope 15 ×). Graphic distributions of positive cells (%) for each antibody and categorical variable in AMEtma group. ^*^Statistical difference between groups comparison. VLVP, vestibular/lingual or vestibular/palatine bone cortical; B, basal bone cortical.
