# Supplementary figures and images for: Expression of DNMTs and H3K9ac in Ameloblastoma and Ameloblastic Carcinoma
Source: Front Oral Health. 2021 Oct 26;2:751162. doi: 10.3389/froh.2021.751162 (PMC8757744; doi:10.3389/froh.2021.751162)

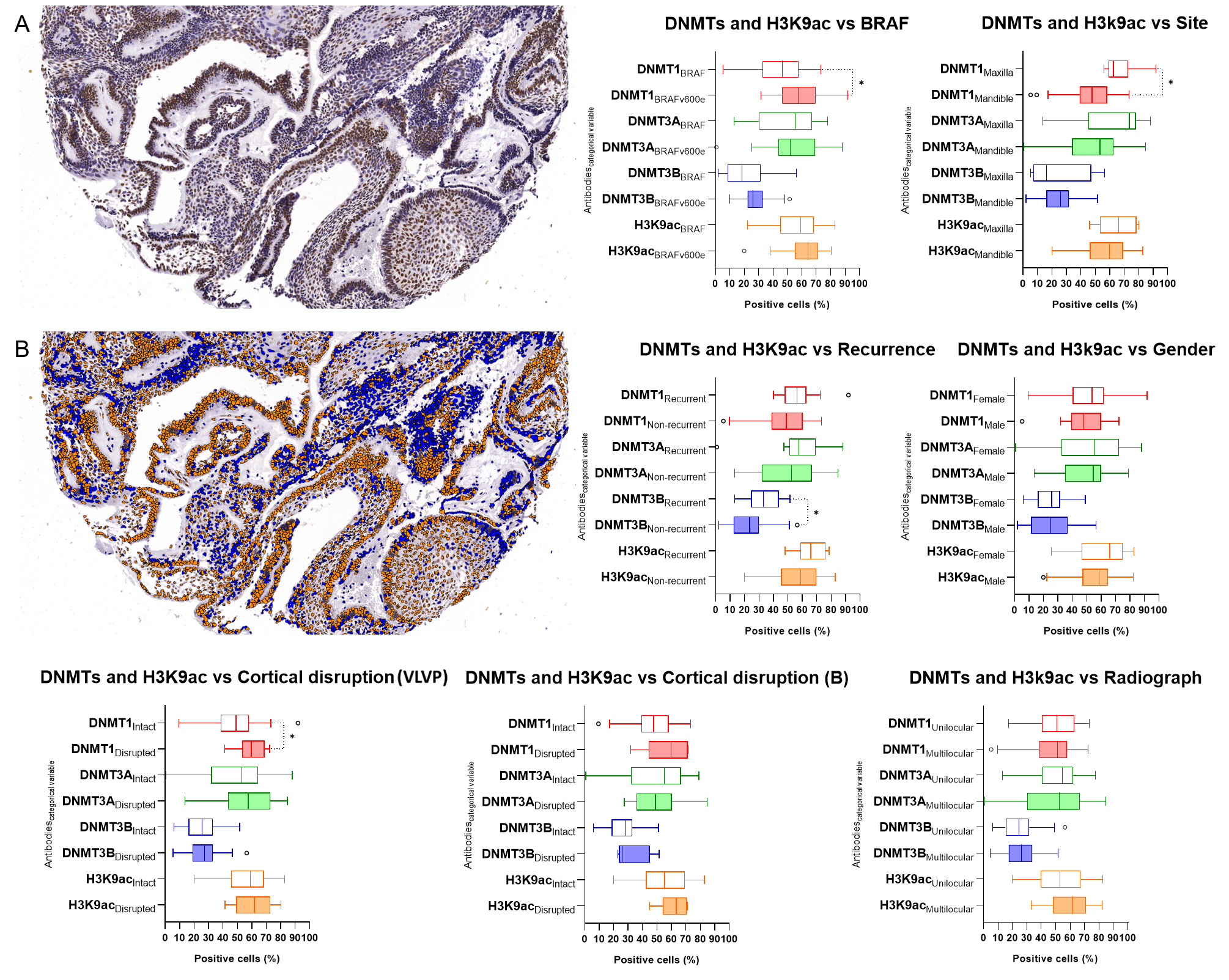

Supplement: Supplementary Figure 1 — A AMEtma core sample before (A) and after application of Nuclear V9 software (B), with DNMT3A reaction (DAB, ScanScope 15 ×). Graphic distributions of positive cells (%) for each antibody and categorical variable in AMEtma group. *Statistical difference between groups comparison. VLVP, vestibular/lingual or vestibular/palatine bone cortical; B, basal bone cortical. [file Image_1.TIF]
